# Supplementary material for: Circulating P2X7 Receptor Signaling Components as Diagnostic Biomarkers for Temporal Lobe Epilepsy
Source: Cells. 2021 Sep 16;10(9):2444. doi: 10.3390/cells10092444 (PMC8467140; doi:10.3390/cells10092444)
Supplement: Supplementary file 1 [file cells-10-02444-s001.zip › Suplementary File/Supplementary Figures.pdf]

Supplementary Figure S1: Additional patient information.

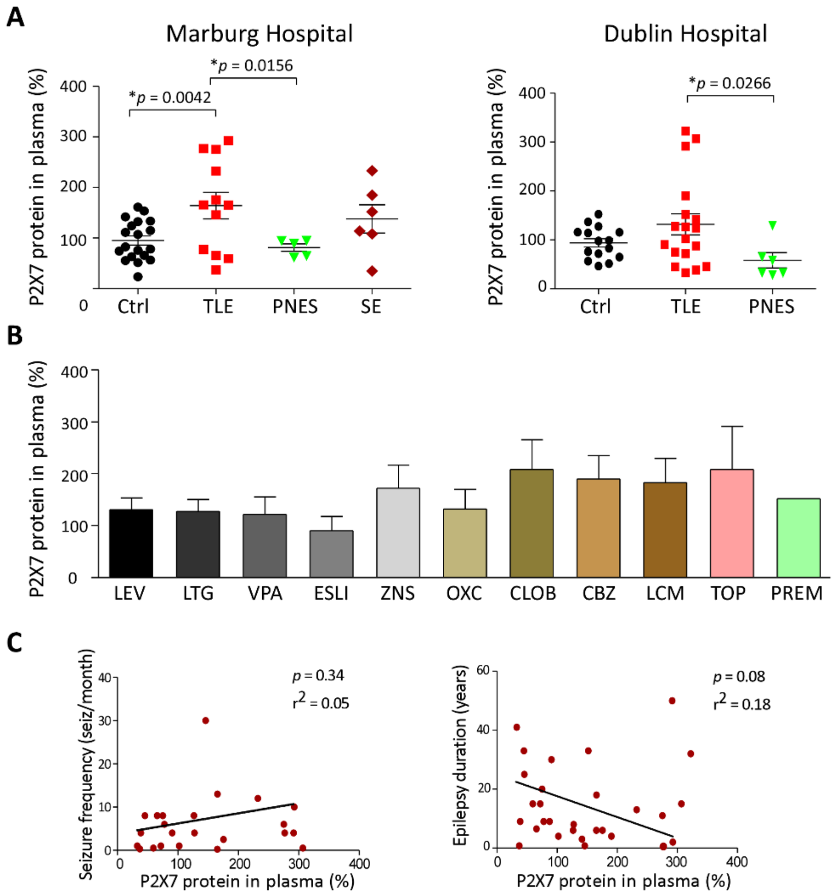

Supplementary Figure S1: (A) Marburg Hospital: Healthy controls (Crtl) (N = 19), TLE patients (N = 12), patients with PNES (N = 5), patients post-status epilepticus (N = 6). Dublin Hospital: Healthy controls (Crtl) (N = 15), TLE patients (N = 18) and patients with PNES (N = 6). ANOVA with post-hoc Fisher correction. Data are given as percentage to control. **(B)** P2X7R plasma concentrations according to treatment with AEDs (N = 16 (LEV, Levetiracetam), 13 (LTG, Lamotrigine), 7 (VPA, Valproic acid), 5 (ESLI, Eslicarbazepine acetate), 5 (ZNS, Zinosamide), 4 (OXC, oxcarbazepine), 4 (CLOB, Clobazam), 3 (CBZ, Carbamazepine), 4 (LCM, Lacosamide), 2 (TOP, Topiramate) and 1 (PREM, Perampanel). **(C)** No significant correlation between P2X7R plasma levels and seizure frequency ($r^2 = 0.05$; $p = 0.34$) and duration of epilepsy ($r^2 = 0.18$, $p = 0.08$). $^*p < 0.05$

Supplementary Figure S2: Cytokine changes in hippocampus and plasma post-status epilepticus measured via cytokine array.

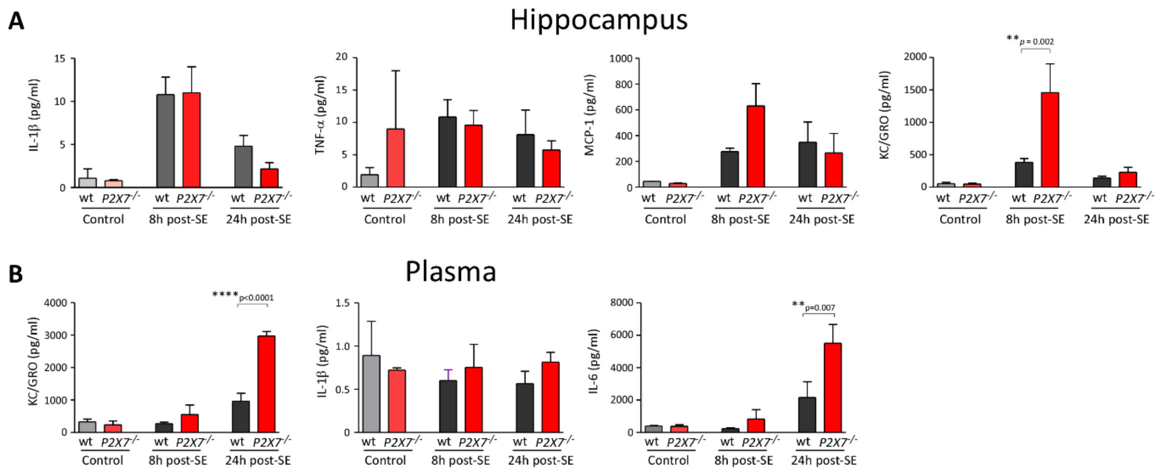

Supplementary Figure S2: (A) Cytokine levels in hippocampal homogenates according to cytokine array results in wt and *P2X7*$^{-/-}$ mice in control conditions and 8 and 24 h post-status epilepticus (SE) for IL-1β, TNF-α, MCP-1 and KC/GRO (N = 2 (wt and *P2X7*$^{-/-}$ control) and 4 (wt and *P2X7*$^{-/-}$ post-SE (8 and 24 h)). **(B)** Cytokine levels in plasma according to cytokine array results in wt and *P2X7*$^{-/-}$ mice in control conditions and 8 and 24 h post-status epilepticus (SE) for KC/GRO, IL-1β and IL-6 (N = 2 (wt and *P2X7*$^{-/-}$ control) and 4 (wt and *P2X7*$^{-/-}$ post-SE (8 and 24 h)).
