# Supplementary material for: Circulating P2X7 Receptor Signaling Components as Diagnostic Biomarkers for Temporal Lobe Epilepsy
Source: Cells. 2021 Sep 16;10(9):2444. doi: 10.3390/cells10092444 (PMC8467140; doi:10.3390/cells10092444)
Supplement: Supplementary file 1 [file cells-10-02444-s001.zip › Suplementary File/Supplementary Table S2.pdf]

Supplementary Table S2: Hippocampal cytokine levels in wt and *P2X7*$^{-/-}$ mice post status epilepticus.

Cytokines/ Chemokines	Hippocampus					
	Controls		Post-status epilepticus (8h)		Post-status epilepticus (24h)	
	wt	*P2X7*$^{-/-}$	wt	*P2X7*$^{-/-}$	wt	*P2X7*$^{-/-}$
**IFN-γ**	Not detected	Not detected	Not detected	Not detected	Not detected	Not detected
**IL-10**	6.74 ± 0.94	6.91 ± 0.04	6.5±3.26	9.37 ± 11.13	2.81 ± 2.36	8.16 ± 4.14
**IL-12p70**	Not detected	Not detected	Not detected	Not detected	Not detected	Not detected
**IL-15**	628.08 ± 205.87	235.7 ± 52.26	275.78 ± 140.4	348.93 ± 267.55	711.54 ± 74.89	432.1 ± 95.08
**IL-17A/F**	214.39 ± 64.67	141.46 ± 3.2	147.56 ± 40.32	243.96 ± 99.84	239.28 ± 33.68	214.48 ± 45.5
**IL-1β**	1.08 ± 1.08	0.8 ± 0.11	10.8 ± 2.86	11 ± 4.26	4.81 ± 1.77	2.15 ± 1.04
**IL-2**	Not detected	Not detected	Not detected	Not detected	Not detected	2.93 ± 4.14
**IL-27p28/IL-30**	30.67 ± 30.67	Not detected	25.5 ± 36.07	30.27 ± 18.08	95.15 ± 41.64	24.72 ± 29.83
**IL-33**	3371.34 ± 913.45	1831.63 ± 794.48	3147.49 ± 321.22	2448.56 ± 498.56	4156.3 ± 1046.85	3448.42 ± 1344.99
**IL-4**	0.69 ± 0.69	Not detected	0.54 ± 0.76	0.22 ± 0.31	1.08 ± 1.52	0.34 ± 0.47
**IL-5**	Not detected	Not detected	0.76 ± 1.07	Not detected	2.66 ± 2.47	3.94 ± 1.57
**IL-6**	125.25 ± 47.17	1553.39 ± 1493.7	272.29 ± 63.1	505.62 ± 144.76	307.86 ± 171.24	391.3 ± 332.8
**IL-9**	586.53 ± 265.49	Not detected	Not detected	321.42 ± 265.12	991.79 ± 513.48	573.12 ± 513.99
**IP-10**	103.07 ± 53.66	53.86 ± 5.16	736.82 ± 374.03	1701.84 ± 767.97	4581.05 ± 1784.95	2656.35 ± 1752.87
**KC/GRO**	54.56 ± 20.02	47.78 ± 14.79	383.34 ± 83.02	1459.54 ± 626.74	141.24 ± 39.53	228.98 ± 108.97
**MCP-1**	45.18 ± 0.39	28.78 ± 4.91	276.1 ± 37.65	630.69 ± 244.7	348.36 ± 221.88	266.45 ± 212.02
**MIP-1α**	117.78 ± 35.41	27.1 ± 27.1	1001.03 ± 187.91	1115.32 ± 240.05	285.35 ± 122.15	262.32 ± 150.86
**MIP-2**	33.59 ± 6.55	12.04 ± 2.79	278.18 ± 49.33	719.95 ± 217.76	96.1 ± 35.83	202.44 ± 133.53
**TNF-α**	1.92 ± 1.1	9 ± 9	10.84 ± 3.77	9.58 ± 3.2	8.1 ± 5.39	5.74 ± 2

Data are given in pg/ml.
