# Supplementary material for: Circulating P2X7 Receptor Signaling Components as Diagnostic Biomarkers for Temporal Lobe Epilepsy
Source: Cells. 2021 Sep 16;10(9):2444. doi: 10.3390/cells10092444 (PMC8467140; doi:10.3390/cells10092444)
Supplement: Supplementary file 1 [file cells-10-02444-s001.zip › Suplementary File/Supplementary Table S3.pdf]

Supplementary Table S3: Plasma cytokine levels in wt and *P2X7*$^{-/-}$ mice post status epilepticus

Cytokines/ Chemokines	Plasma					
	Controls		Post-status epilepticus (8h)		Post-status epilepticus (24h)	
	wt	*P2X7*$^{-/-}$	wt	*P2X7*$^{-/-}$	wt	*P2X7*$^{-/-}$
**IFN-γ**	0.75 ± 0.42	0.47 ± 0.32	0.35 ± 0.15	0.35 ± 0.07	0.73 ± 0.2	0.79 ± 0.32
**IL-10**	33.33 ± 4.09	21.87 ± 1.29	28.5 ± 4.82	61.64 ± 27.97	459.62 ± 194.05	1700.38 ± 486.1
**IL-12p70**	64.07 ± 64.07	Not detected	Not detected	29.65 ± 17.79	30.38 ± 8.16	41.74 ± 7.09
**IL-15**	39.37 ± 3.2	75.75 ± 34.35	16.99 ± 9.82	16.99 ± 9.83	63.47 ± 7.02	72.55 ± 10.41
**IL-17A/F**	Not detected	Not detected	Not detected	Not detected	36.54 ± 18.73	49.32 ± 18.15
**IL-1β**	0.89 ± 0.4	0.72 ± 0.03	0.6 ± 0.13	0.75 ± 0.27	0.57 ± 0.14	0.81 ± 0.11
**IL-2**	0.65 ± 0.21	1.71 ± 0.42	0.9 ± 0.52	1.21 ± 0.27	2.31 ± 0.59	2.32 ± 0.49
**IL-27p28/IL-30**	10.64 ± 7.19	2.09 ± 2.09	7.61 ± 4.02	2.25 ± 2.25	63.03 ± 56.24	16.79 ± 7.72
**IL-33**	0.86 ± 0.86	9.98 ± 9.37	2.63 ± 1.54	1.44 ± 0.68	0.97 ± 0.33	1.03 ± 0.62
**IL-4**	1.25 ± 1.19	0.05 ± 0.05	0.1 ± 0.06	0.7 ± 0.47	0.03 ± 0.03	0.04 ± 0.04
**IL-5**	9.88 ± 0.62	11.61 ± 6.45	17.28 ± 11.28	13.64 ± 8.54	6.75 ± 3.08	4.25 ± 1.14
**IL-6**	397.99 ± 28.59	376.01 ± 92.25	235.85 ± 39.54	828.59 ± 573.32	2156.3 ± 975.16	5507.76 ± 1160.05
**IL-9**	Not detected	Not detected	Not detected	Not detected	Not detected	Not detected
**IP-10**	427.4 ± 136.16	400.21 ± 255.16	235.78 ± 14.19	142.55 ± 36.57	133.58 ± 31.23	216.61 ± 82.6
**KC/GRO**	325.1 ± 85.06	233.63 ± 118.93	267.5 ± 51.42	551.66 ± 293.66	964.6 ± 242.61	2971.14 ± 141.66
**MCP-1**	69.53 ± 25.72	36.67 ± 11.66	39.67 ± 4.97	39.9 ± 9.22	52.03 ± 10.83	222.89 ± 54.12
**MIP-1α**	1.27 ± 1.27	Not detected	0.72 ± 0.72	13.85 ± 8.01	69.65 ± 28	93.02 ± 14.4
**MIP-2**	33.94 ± 7.98	24.84 ± 0.35	50.25 ± 2.05	241.35 ± 119.28	1951.63 ± 625.03	2650.89 ± 575.42
**TNF-α**	32.23 ± 5.76	15.92 ± 2.13	9.71 ± 0.63	59.75 ± 30.91	518.69 ± 222.41	484.38 ± 115.68

Data are given in pg/ml.
